# Phylogeographic analysis of *Staphylococcus nepalensis* reveals global occurrence of antimicrobial-resistant lineages carrying the *sal*(E) resistance gene

**DOI:** 10.1007/s11033-026-12601-4

**Published:** 2026-09-04

**Authors:** Johana Becerra, Isabella Gaião, Roberta Almeida Vincenzi, Davi Gabriel Salustiano Merighi, Felipe Vásquez-Ponce, Santiago Justo Arevalo, Jesus Pariona, Gregory Melocco, Fernanda Esposito, Fabio Rodrigues, Nilton Lincopan

**Affiliations:** 1 Department of Microbiology, Institute of Biomedical Sciences, University of São Paulo, São Paulo, Brazil; 2 One Health Brazilian Resistance Project (OneBR), São Paulo, Brazil; 3 Antimicrobial Resistance Institute of São Paulo (ARIES), São Paulo, Brazil; 4 Department of Biochemistry, Institute of Chemistry, University of São Paulo, São Paulo, Brazil; 5 Facultad de Ciencias Biológicas, Universidad Ricardo Palma, Santiago de Surco, Peru; 6 Department of Clinical and Toxicological Analysis, School of Pharmacy, University of São Paulo, São Paulo, Brazil

**Keywords:** Genomic surveillance, One health, Coagulase-negative staphylococcus, Hypersaline lagoon, Brazil

## Abstract

**Background:**

*Staphylococcus nepalensis* is an emerging species first described in 2003 from the respiratory tract of goats in Nepal. We report the identification of *S. nepalensis* of a hypersaline lagoon in Brazil, along with in-depth phylogeographical and resistome analysis of publicly available genomes.

**Methods and results:**

During a local survey from hypersaline aquatic environments in Rio de Janeiro, Brazil, two staphylococcal strains were recovered, designated as COLB and AM1. These isolates were subjected to antimicrobial susceptibility testing, genomic sequencing, and comprehensive phylogenomic analyses. Genomic analysis confirmed the taxonomic identity of COLB and AM1 as *S. nepalensis.* Both isolates harbored the *sal*(E) conferring resistance to pleuromutilins and streptogramin A, whereas *tet*(K) conferring to tetracyclines. Additionally, AM1 carried *lnu*(A), consistent with the reduced susceptibility to clindamycin (MIC = 2 µg/mL) relative to COLB. Genes associated with arsenic and copper tolerance, and the replicons *rep7a* and *rep19c*, were confirmed. Phylogenomic analysis indicated that COLB and AM1 were clonally related (1 cgSNP-difference) but distinct from global isolates. Phylogeographic analysis revealed wide geographic occurrence, with some lineages carrying *blaZ* and *mecA* associated with beta-lactamase production and methicillin resistance, respectively. Strikingly, *sal*(E) is conserved across all *S. nepalensis* genomes.

**Conclusions:**

The findings confirm the presence of *S. nepalensis* in South America as early as 2016 and documented among available genomes from environmental, human, and animal-associated sources. Furthermore, reveal the circulation of some lineages carrying clinically relevant antimicrobial genes, underscoring the importance of accurate species identification and continuous genomic surveillance and potential One Health relevance.

**Supplementary Information:**

The online version contains supplementary material available at https://doi.org/10.1007/s11033-026-12601-4.

## Introduction

The genus *Staphylococcus* comprises Gram-positive, cocci-shaped, facultative anaerobic bacteria, catalase-positive, non-motile, and non-spore-forming [1]. This diverse genus comprises environmental species as well as opportunistic pathogens of humans and animals, with at least 73 validly named species listed in the *List of Prokaryotic Names with Standing in Nomenclature* [2]. The clinical importance of *Staphylococcus* is due to its capacity to acquire and disseminate mobile genetic elements carrying antimicrobial resistance (AMR) determinants, enabling rapid adaptation across hosts and settings [3].

Among the staphylococci, *Staphylococcus aureus*, a coagulase-positive species (CoPS), remains the most important pathogen associated with a wide range of community, hospital-acquired, and recently livestock-acquired infections [1, 3, 4]. In addition, coagulase-negative staphylococci (CoNS) have emerged over recent decades as significant opportunistic pathogens. Notably, species such as *S. epidermidis*, *S. lugdunensis*, *S. saprophyticus*, *S. haemolyticus* [5–7] and *S. nepalensis* have been implicated in clinically relevant infections including bacteremia [8], and urinary tract infections [9].

*S. nepalensis* is a CoNS, first described in 2003 from the respiratory tract of goats in the Himalayan region of Nepal [10]. Nevertheless, retrospective evidence indicates that this species had been circulating earlier. Isolates recovered in the 1960s in the United Kingdom from environmental and animal samples were initially classified as *S. saprophyticus* and *S. xylosus* (GenBank: UHDY00000000.1 and UHEJ00000000.1, respectively) but were reidentified as *S. nepalensis* in 2020. Since then, *S. nepalensis* has been reported in diverse geographic regions and hosts, including humans, highlighting its potential as both a nosocomial and zoonotic pathogen.

In Brazil, *S. nepalensis* has been detected in diverse animal-associated niches, including the oral commensal microbiota of domestic cats, where it has been identified as a reservoir of transferable antimicrobial resistance (AMR) determinants [11, 12]. Additionally, it has been recovered from feline urinary tract infections [13] and, more recently, isolated from the oral cavity of a captive snake [14].

This study reports the detection and characterization of *Staphylococcus nepalensis* isolates from a disturbed hypersaline lagoon in Rio de Janeiro, Brazil. Phylogeographic analysis based on publicly available genomes, combined with whole-genome sequencing (WGS) of our environmental isolates, documented among environmental, human, and animal-associated sources, and resistome diversity of this species. These findings contribute to the current understanding of *S. nepalensis* as a potential emerging One Health pathogen. Furthermore, comparative genomics showed the *sal*(E) gene was conserved across all *S. nepalensis* genomes analyzed, consistent with a vertically inherited species-associated determinant.

## Materials and methods

### Bacterial isolation and identification

Water and sediment samples were collected from a disturbed hypersaline lagoon system of Araruama (Brejo do Espinho; 22°56’03.4"S, 42°14’20.6"W), Rio de Janeiro, Brazil, and transported in sterile Whirl-Pak^®^ bags at 4 °C. Microorganisms were isolated using a modified Luria–Bertani (LB) agar medium supplemented with 70 g L^-1^ NaCl (saline LB). The samples were suspended in an 80 g L^-1^ NaCl solution and spread onto saline LB agar plates. Following incubation at 30 °C for 48 h, colonies were purified through repeated streaking. Pure cultures were stored in 20% (v/v) glycerol at -80 °C for subsequent analyses. Initial identification was performed by MALDI-TOF MS (Matrix-Assisted Laser Desorption/Ionization Time-of-Flight Mass Spectrometry; Bruker, Daltonics).

### Antimicrobial susceptibility profile

The antimicrobial susceptibility profile of the isolates was determined by the disk-diffusion method on Mueller–Hinton agar (Difco) following standard guidelines [15, 16]. The antimicrobials tested (µg/disk) included: penicillin (10 units), cefoxitin (30), doxycycline (30), clindamycin (2), rifampicin (30), ciprofloxacin (5), chloramphenicol (30), mupirocin (200), linezolid (30), lincomycin (2), and oxacillin (1). In addition, the minimum inhibitory concentrations (MICs) of vancomycin for COLB and AM1 isolates were determined by broth microdilution method in accordance with ISO 20,776 [15, 16]. Briefly, the bacterial inoculum was adjusted to a 0.5 McFarland standard and diluted 1:10 in Mueller-Hinton broth (Becton, Dickinson, France). For clindamycin and quinupristin-dalfopristin, the E-test (BioMérieux and Oxoid) was used. Interpretative criteria were based on the EUCAST and CLSI guidelines [15, 16]. The clinical strain *S*. *aureus* BAA 1556 was used as a quality-control strain. Antimicrobial disks were obtained from Cefar Diagnóstica Ltda (São Paulo, Brazil) or purchased from Liofilchem (Roseto D.A., Italy). Multidrug resistance (MDR) was defined as nonsusceptibility to at least one agent in three or more antimicrobial categories [17].

### Whole-genome sequencing (WGS) analysis

Genomic DNA from COLB and AM1 was extracted using the PureLink Quick Gel Extraction Kit (Life Technologies, USA). Paired-end libraries were prepared with the Nextera DNA Flex Library Preparation Kit (Illumina Inc., UK) following the manufacturer’s instructions and sequenced on the Illumina NextSeq platform (Illumina Inc., USA). Raw reads were quality-trimmed with Trimmomatic (https://github.com/usadellab/Trimmomatic) and assembled *de novo* with SPAdes (https://github.com/ablab/spades).

All publicly available *Staphylococcus nepalensis* genomes (Taxon ID: 214473) were retrieved from NCBI using the Datasets CLI tool v18.29.1. In cases where duplicate genomes shared the same BioSample accession, the latest version was included, prioritizing RefSeq (GCF) accessions over GenBank (GCA) accessions. Genome quality was assessed using CheckM2 v1.1.0 (https://github.com/chklovski/CheckM2) and QUAST v5.2.0 (https://github.com/ablab/quast). Genomes were excluded from downstream analyses if they exhibited predicted completeness of ≤ 95%, contamination of ≥ 5%, more than 100 contigs, or more than 500 ambiguous bases (Ns). Species identity was confirmed using FastANI v.1.34 (https://github.com/ParBLiSS/FastANI), with a threshold of ≥ 95% ANI relative to the *S. nepalensis* reference strain JS11 (CP017466.1). Genome annotation was performed using Bakta v1.12.0 (https://github.com/oschwengers/bakta).

Pangenome reconstruction and core-genome alignment were performed using Panaroo v1.7.0 (https://github.com/gtonkinhill/panaroo). Genes detected in 99–100% of isolates were designated part of the core-genome. Putative recombinant regions, identified as clusters of elevated single nucleotide polymorphisms (SNPs) within the core-genome alignment, were detected and masked using Gubbins v2.5.1 [18] (https://github.com/nickjcroucher/gubbins). The proportion of SNPs attributable to recombination was determined from the per-branch statistics output. The relative rate of recombination to mutation (r/m ratio) was estimated using ClonalFrameML v1.20 (https://github.com/xavierdidelot/ClonalFrameML). Recombination parameters, including the mean recombinant tract length (δ) and the mean divergence of imported sequences (ν), were estimated, with uncertainty assessed through 100 simulations (-emsim 100). The relative contribution of recombination to genetic diversification (r/m) was calculated as r/m = (R/θ) × δ × -ν [19].

Core-genome SNPs (cgSNPs) were extracted from the Gubbins recombination-filtered alignment using SNP-sites v2.5.1 (https://github.com/sanger-pathogens/snp-sites). A pairwise SNP distance matrix was calculated using SNP-dists v1.2.0 (https://github.com/tseemann/snp-dists). Phylogenetic reconstruction was performed with IQ-TREE v3.1.2 (https://github.com/iqtree/iqtree3) using the GTR + G substitution model on the recombination-filtered SNP alignment, with constant site correction applied via the -fconst option. Branch support was then assessed using 1,000 ultrafast bootstrap replicates (UFBoot2). Nodes with bootstrap values ≥ 95% were considered well-supported [20]. Finally, tree topology and metadata were visualized using iTOL v6 (https://itol.embl.de).

Antimicrobial resistance genes were identified using AMRFinderPlus v4.2.7 with the NCBI database (2026-05-15.1; https://github.com/ncbi/amr) using *S. aureus* as the reference organism for point mutation detection. The --plus option was enabled to extend the search to include stress-response genes, heavy-metal resistance determinants, and biocides. Resistance genes were additionally identified using RGI with the Comprehensive Antibiotic Resistance Database resistance genes (CARD) v3.2.7 (https://card.mcmaster.ca/analyze/rgi).

Virulence-associated genes were predicted using VirulenceFinder v2.0.1 (https://github.com/genomicepidemiology/virulencefinder) with the *S. aureus*-specific databases (*s.aureus*_toxin, *s.aureus*_exoenzyme, *s.aureus*_hostimm) and Abricate with the Virulence Factor Database v1.0.1 (VFDB) (https://www.mgc.ac.cn/VFs/). Plasmid replicons were identified using PlasmidFinder v2.2.0 (https://github.com/genomicepidemiology/plasmidfinder) with the Gram-positive database. For all databases, thresholds of ≥ 90% nucleotide identity and ≥ 60% gene coverage were applied to reliably detect known antimicrobial resistance and virulence genes while allowing identification of allelic variants and partial gene sequences in draft genome assemblies [21]. Moreover, for publicly available genomes, predicted genes were considered indicators of potential resistance capacity rather than as confirmed resistance phenotypes.

### In silico modeling and alignment of Sal(E) sequences

The three-dimensional structure of Sal(E) from *S. nepalensis was predicte*d using AlphaFold*3* (WP_096809342.1; https://alphafoldserver.com). Structural alignment was performed to identify the Sal(E) region corresponding to the α-helices of Sal(B) known to interact with the 50 S ribosomal subunit in the experimentally determined ribosome-bound complex (DOI:https://doi.org/10.1093/nar/gkac058; PDB: 7P48). Structural visualization and analyses were conducted using ChimeraX v1.18.0. (https://www.rbvi.ucsf.edu/chimerax).

Conservation of the *sal*(E) gene across *S. nepalensis* genomes was assessed by extracting protein sequences annotated as *sal*(E) from Bakta annotation. Multiple sequence alignment was conducted using MAFFT v7.525 (https://mafft.cbrc.jp/alignment/software/) with the L-INS-i algorithm and pairwise sequence identity was calculated from the resulting alignment. Representative sequences were selected with CD-HIT (https://github.com/daugherty-lab/CD-hit), and five representative sequences were aligned with Clustal Omega v.1.2.4 (www.ebi.ac.uk/jdispatcher/msa/clustalo). The AlphaFold3-predicted structural model of Sal(E) (WP_096809342.1) was used as a reference to map secondary structure onto the alignment using ESPrit v3.0 (https://espript.ibcp.fr). The annotated alignment was visualized in Jalview v2.11.3.0 (https://www.jalview.org).

### Genetic context of Sal(E) in *Staphylococcus nepalensis*

Gene neighborhood conservation analysis for the sal(E) locus in the *S*. *nepalensis* strains was conducted using the comparative genomics tool ROTIFER v.1.0 (Rapid Open-source Tools and Infrastructure for Data Exploration and Research; https://github.com/leepbioinfo/rotifer). The nucleotide sequences flanking the *sal*(E) gene were extracted and annotated using the Pfam database (https://pfam.xfam.org/). Domain assignments were filtered based on score and e-value thresholds to retain only high-confidence annotations. The resulting domain organization and gene neighborhood architecture for each locus were visualized and plotted using the gggenes v0.5.0 package in RStudio v.2025.05.0.

### Virulence potential in the *Galleria mellonella* larvae model

The virulence of *S. nepalensis* strains COLB and AM1 was tested in *Galleria mellonella* larvae. Groups of 10 larvae (230–280 mg) were injected with 10 µL of 2.5 × 10^8^ CFU/mL in PBS. A control group received 10 µl PBS to rule out death by physical trauma. The clinical strain *S. aureus* BAA 1556 (USA 300) was used as a virulent control. Post-injection, larvae were incubated at 37 °C. Survival was recorded hourly for 96 h. Three biological replicates were done, with 10 larvae per strain. Survival curves were plotted using the Kaplan-Meier method, and statistical significance was determined by the log-rank test (*p* < 0.05; Origin Software, United States).

All bioinformatics analyses were performed using a custom pipeline developed entirely in-house [22]. Complete scripts, software versions, parameters, and step-by-step documentation are publicly available in the GitHub repository -(https://github.com/zbecerra/Phylogeographic-analysis-of-S.-nepalensis-carrying-the-intrinsic-sal-E-resistance-gene), and permanently archived at Zenodo (https://doi.org/10.5281/zenodo.20645567).

## Results and discussion

In this study, we conducted a genomic surveillance investigation between 2016 and 2018 by screening microbial mat samples in an anthropogenically impacted hypersaline lagoon in Rio de Janeiro, Brazil. From these samples, two staphylococcal strains were isolated, designated as COLB and AM1, recovered in 2016 and 2018, respectively. Both isolates were initially identified by MALDI-TOF MS and subsequently subjected to Whole-genome sequencing (WGS). Species identity of COLB (GenBank: JBDFMR000000000.1) and AM1 (GenBank: JBEGDQ000000000.1) were confirmed by average nucleotide identity (ANI) analysis against the *S. nepalensis* reference strain J11 (GenBank: CP017466.1), yielding ANI values of 99.60% and 99.69%, respectively. Consistently, all publicly available genomes included in this study exhibited ANI values ≥ 98.99%, above the accepted ≥ 95% threshold for species delineation [23], confirming their classification as *S. nepalensis* (Table S1).

**Fig. 1:**
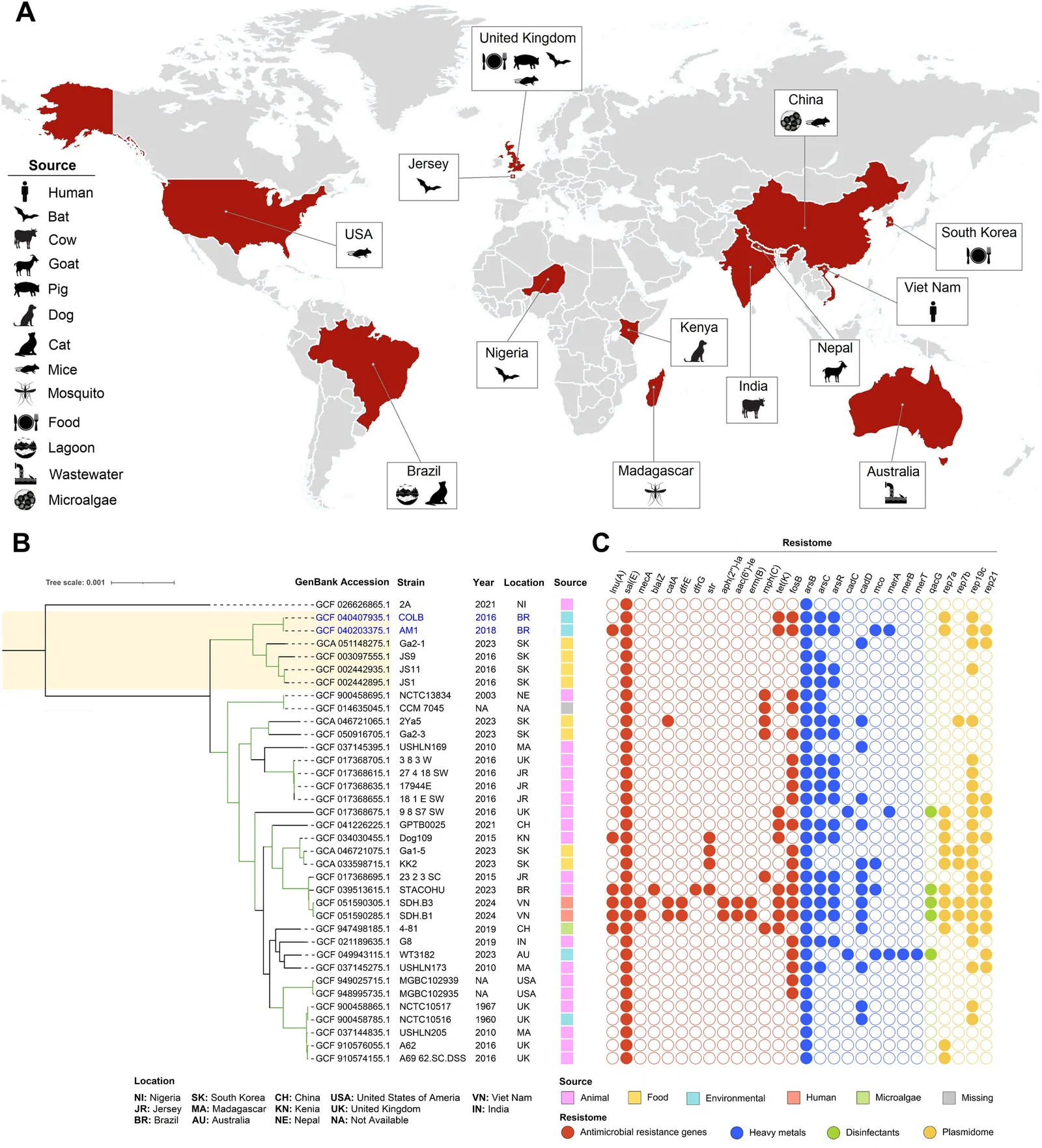
Global detection and genomic characterization of 36 publicly available *S. nepalensis* genomes. (**A)** Geographic occurrence of *S. nepalensis* reports worldwide, indicating the locations and sources from which the species has been identified through whole-genome sequencing (WGS). (**B)** Core-genome SNP-based phylogenomic analysis of *S. nepalensis* worldwide. Maximum-likelihood phylogenetic tree inferred with IQ-TREE v3.1.2 under the GTR + G substitution model using 20,043 recombination-corrected cgSNPs. Nodes with 100% bootstrap support are highlighted in green. Brazilian isolates COLB and AM1 are highlighted in blue. **(C**) Distribution of resistome profiles according to the main categories: antimicrobial resistance genes (red), heavy metal resistance genes (blue), disinfectant resistance genes (green), and plasmid replicons (yellow)

Comparative phylogenomic analysis of 36 publicly available *S. nepalensis* genomes revealed the detection of this species across diverse ecological niches, including environmental, food, animal, and human sources (Fig. 1A and Table S1). However, these observations reflect the currently available genomic data rather than the true global distribution of the species and do not reflect the worldwide prevalence. In Brazil, *S. nepalensis* was first reported in 2020, as part of the commensal oral microbiota of domestic cats [11], later in 2024 from a urine infection sample [13], and more recently from the oral cavity of a captive snake [14]. Our phylogeographical analysis suggests that this species had already been circulating in Brazil since at least 2016, extending the timeline of *S. nepalensis* in South America but also emphasizing its ecological versatility and the need for broader One Health surveillance of this species.

The core-genome SNP (cgSNP)-based phylogenomic analysis, based on 2,348 core genes and 20,043 SNP positions, yielded a well-resolved maximum-likelihood tree with the majority of nodes supported by bootstrap values of 100% highlighted in green, indicating high confidence in the overall tree topology [20] (Fig. 1B). Moreover, COLB and AM1 showed a clonal relationship characterized by only a single cgSNP difference over a two year sampling interval, below the clonality threshold of 15 cgSNPs within a period of six months proposed for meticillin-resistant *Staphylococcus aureus* [24], supporting their classification as clonally related isolates. Furthermore, COLB and AM1 clustered with strains from Korean fermented foods (JS9, JS11, JS1; GenBank: CP017459.1, CP017460.1, CP017466.1, respectively), differing by at least 1,648 cgSNPs and more than 3,491 cgSNPs from other globally distributed strains, indicating no evidence of clonal relatedness (Table S2).

Phenotypic antimicrobial susceptibility testing confirmed that both COLB and AM1 were resistant to quinupristin-dalfopristin, lincomycin, tetracycline, and doxycycline, with quinupristin-dalfopristin MICs of 3 µg/mL, and 6 µg/mL, respectively. In addition, AM1 exhibited intermediate susceptibility to clindamycin (MIC = 2 µg/mL), while both isolates remained susceptible to all other antimicrobials tested. These phenotypic profiles were consistent with the genomic resistome, which identified *sal*(E) that contribute to the susceptibility reduction to quinupristin-dalfopristin [25], and *tet*(K), conferring efflux-mediated resistance to tetracycline and doxycycline [26]. Moreover, AM1 harbored *lnu*(A), encoding a lincosamide nucleotidyltransferase that confers resistance to lincomycin and clindamycin [27], likely contributing to reduced susceptibility clindamycin MIC relative to COLB. Despite the absence of *lnu*(A) in COLB, the lincomycin resistance observed in both isolates may reflect a species-specific contribution of *sal*(E) to lincosamide protection in the native S. *nepalensis* ribosomal context.

Beyond antimicrobial resistance genes, COLB and AM1 revealed additional adaptive features with ecological relevance as heavy-metal tolerance genes *arsR*, *arsB*, and *arsC*, components of the arsenic resistance operon originally described in *S. aureus* plasmid pI258 [28]. These genes confer tolerance to arsenate [As(V)], arsenite [As(III)], and antimonite [Sb(III)], frequently encountered in anthropogenically impacted aquatic environments [29]. Notably, AM1 present additionally acquire *mco* gene, associated with copper tolerance and oxidative stress protection in *S. aureus* [30], as well as *merA* which encodes a mercuric reductase previously described on the *S. aureus* plasmid pI258 [31].

Analysis of the mobilome revealed that both COLB and AM1 carried the plasmid replicons *rep7a* and *rep19c*, whereas AM1 additionally harbored *rep21*, previously associated with plasmid carrying *Inu*(A) [32]. Moreover, the *rep7* and *rep21* are among the most prevalent plasmid replicon groups identified in staphylococci from human, animal, and food sources [32] (Fig. 1C). The additional plasmid replicons detected in AM1 relative to COLB are consistent with possible involvement of mobile genetic elements, although the timing and mechanism of their acquisition cannot be determined from the present data.

Moreover, both isolates exhibited a non-virulent phenotype in the *Galleria mellonella* infection model, with 100% larval survival maintained throughout the 96-hour observation period. Consistent with these findings, no known virulence-associated genes were detected in any of the 36 analyzed genomes using either VirulenceFinder or Abricate with the VFDB database.

In silico resistome analysis predicted for publicly available genomes globally distributed *S. nepalensis* isolates, revealed a heterogeneous repertoire of antimicrobial resistance genes, including *blaZ* and *mecA* for β-lactam antibiotics; *lnu*(A) for lincosamides; *tetK* for tetracyclines; *cat*A for chloramphenicol; *dfrE* and *dfrG* for trimethoprim; *aac(6’)-Ie* for aminoglycosides; and *mphC* and *ermB* for macrolides, among others. Heavy-metal resistance genes, including *arsR*, *arsB*, *arsC*, *cadD*, *mco*, *merA*, *merB*, and *merT*, were widely distributed, indicating adaptation to polluted environments [28, 29]. The presence of *qacG* indicated resistance to quaternary ammonium compounds, commonly used as disinfectants, and plasmid replicons *rep7a*, *rep7b*, *rep19c*, and *rep21* reflected a dynamic mobilome with high dissemination potential (Fig, 1 C and Table S1).

Importantly, the *mecA* gene encoding the penicillin-binding protein PBP2a, which confers methicillin resistance in staphylococci, was recently detected in two *S. nepalensis* strains from human infections in 2024 in Viet Nam. The correct classification and accurate identification are fundamental for clinical and epidemiological purposes. Misidentification of this species could lead to an underestimation of its clinical relevance, improper diagnosis, and delay the adoption of effective antimicrobial therapies. Accurate taxonomic identification is crucial for differentiate *S. nepalensis* from other CoNS, which often exhibit different antimicrobial resistance profiles and pathogenic potential.

Bioinformatic resistome prediction across all publicly available *S. nepalensis* genomes consistently identified *sal*(E) in all analyzed isolates, consistent with a conserved vertically inherited species-associated determinant [33]. Alignment of five representative Sal(E) protein sequences from *S. nepalensis* revealed high overall conservation, with only seven amino acid substitutions (Cys179, His229, Arg312, His386, His431, Asp450, Ile473) and two deletions at residue positions 60 and 505, corresponding to a sequence conservation of 97.8% across the alignment (531/543 positions conserved) (Fig. S1). Notably, none of these substitutions occurred within the antibiotic resistance domain loop, the functionally critical region responsible for antibiotic displacement. However, the functional impact of the observed sequence conservation across all lineages remains to be experimentally validated.

**Fig. 2:**
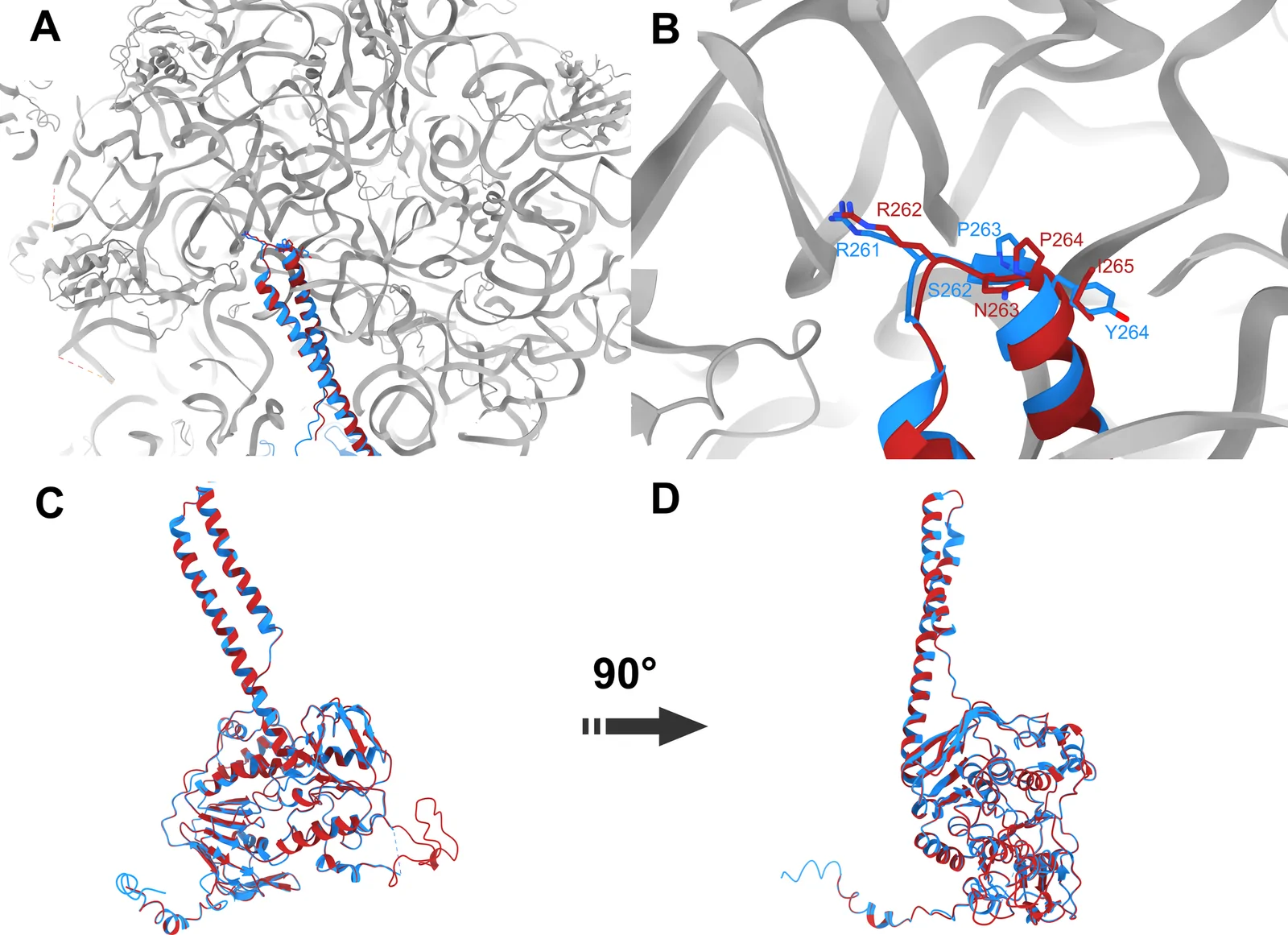
Structural characterization of Sal(E) from *S. nepalensis.* (**A)** Structural comparison of the *S. aureus* 50 S ribosomal subunit (grey) in complex with Sal(B) (PDB: 7P48, blue) and the AlphaFold predicted for Sal(E) from *S. nepalensis* (WP_096809342.1; red). (**B)** Structural comparison of Sal(E) from with Sal(B). Key residue positions are labelled for both proteins, highlighting amino acid substitutions at positions between homologs. (**C, D)** Tertiary structures, represented as cartoon models. Sal(E) aligned with the reference Sal(B) structure from two orientations rotated 90° relative to each other. Structural visualizations were generated using ChimeraX v1.10

**Fig. 3:**
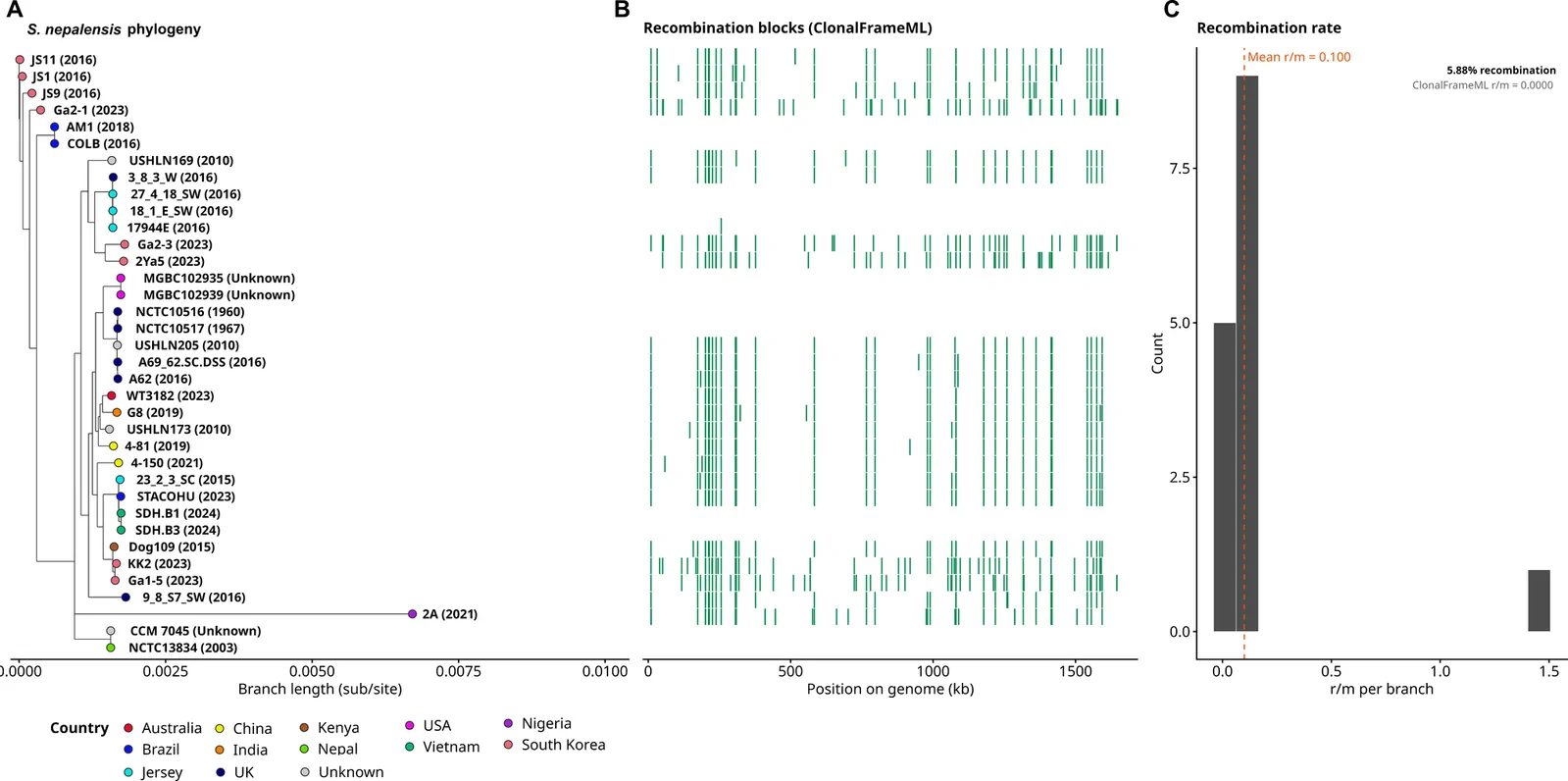
Recombination analysis of *S. nepalensis* based on 36 publicly available genomes. **(A)** Recombination-corrected maximum-likelihood phylogeny inferred from the Gubbins tree, with branch lengths representing evolutionary distances in substitutions per site after conversion from recombination-masked SNP counts. Colored circles at tip nodes indicate a country and year isolation. **(B)** Genomic distribution of recombination blocks detected by ClonalFrameML across the *S. nepalensis*. Each horizontal row corresponds to one isolate aligned to the phylogeny in panel A, and green vertical bars indicate the genomic position. **C.** Distribution of per-branch recombination-to-mutation ratios (r/m), showing an overall low contribution of recombination and a predominantly clonal population structure

In silico structural prediction of Sal(E) using AlphaFold3 generated a high-confidence model (pTM = 0.8), revealing the positioning in complex with the bacterial 50 S ribosomal subunit (grey) (Fig. 2A). The interhelical loop tip of Sal(B) is characterized by an arginine, R261 followed by S262, and Y264, while the equivalent region in Sal(E) also present an arginine (R262), followed by a polar residue N263, a proline P264 and a hydrophobic residue I265 [25]. Those residues in Sal(E) could also mediate the blocking of the antibiotic binding as in Sal(B), although confirming experiments are needed to test this hypothesis (Fig. 2B). Furthermore, structural comparison revealed that Sal(E) adopts the canonical ABC-F fold, with an overall architecture similar to Sal(B) conserving the global protein topology (Fig. 3C and D).

Comparative genomic analysis of *S. nepalensis* revealed that *sal*(E) is consistently located within a conserved chromosomal region. Upstream genes include cysteine desulfurase (*iscS*/*nifS*), *mnmA* (tRNA modification enzyme), tetratricopeptide repeat (TPR) proteins, and *recD2* or related helicases. Downstream region typically encodes alanine-tRNA ligase (*alaS*), Holliday junction resolvase (*ruvX*), aspartate-tRNA ligase (*aspS*), and 6 S RNA (*ssrS*) (Fig. S2). This conserved organization suggests a cluster related to tRNA modification, translation fidelity, and DNA repair. Similarly, *sal*(A) detected in *S. sciuri*, is localizated in housekeeping genes regions supporting an ancient evolutionary origin, and limited potential for horizontal gene transfer and dissemination [34]. Furthermore, sal-type genes appear to represent an evolutionarily conserved resistance determinants across multiple *Staphylococcus* species. The congruence between Sal protein phylogeny and species phylogeny supports their predominantly vertical inheritance [25].

Recombination detection using Gubbins identified 211 recombination blocks across the core-genome alignment of 2,348 core genes with 1,677,386 total positions. Analysis indicated that recombination accounted for 5.88% of detected SNPs, consistent with a predominantly clonal population structure [18] (Fig. 3B). After recombination masking, the alignment retained 20,043 variable positions, which were extracted using snp-sites and used as input for maximum-likelihood phylogenetic reconstruction with IQ-TREE (Fig. 3A). Importantly, recombination analysis confirmed that the clonal relationship between COLB and AM1 was unaffected by recombination masking, with identical pairwise distances obtained both before and after Gubbins filtering, confirming the robustness of the single SNP difference.

Estimation of recombination parameters using ClonalFrameML yielded an R/θ of 3.97 × 10^5^, a mean recombinant tract length (δ) of 97.58 bp, and a mean import divergence (ν) of 2.09 × 10^− 4^, resulting in an r/m ratio of 8.09 × 10^− 7^ (Fig. 3A-C). These results indicate that recombination contributes minimally to genetic diversification, with point mutations occurring far more frequently. The short mean tract length (97.58 bp) is reflected in recombination blocks as narrow discrete marks rather than broad segments, consistent with localized recombination events distributed across the genome rather than large-scale chromosomal replacements (Fig. 3B). Moreover, the mean r/m per branch of 0.100 further supports the predominantly clonal nature of *S. nepalensis*, with 35 of 36 branches exhibiting r/m values below 0.16 (Fig. 3C). The single outlier branch showed elevated recombination corresponding to the strain Ga1-5 isolated from fermented korean food with a r/m of 1.45, corresponding to 27 total SNPs. Nevertheless, the general population structure described is consistent with that reported for coagulase-negative staphylococci [35] (Fig. 3C).

Beyond the core genome, the *S. nepalensis* pangenome comprised 2,348 core genes, 485 shell genes, and 1,880 cloud genes, totalling 4,753 genes across the 36 analyzed genomes. The large accessory genome fraction corresponds to 51%, consistent with an open pangenome structure [33], indicating ongoing gene acquisition through horizontal transfer. These findings indicate that *S. nepalensis* possesses a flexible accessory genome that may contribute to adaptation across diverse hosts and ecological niches. However, these conclusions are based on a limited and geographically biased dataset of 36 publicly available genomes, which reflects opportunistic genome availability rather than systematic sampling. Expanding genomic surveillance and increasing the representation of isolates from under sampled regions and sources will be critical for a more comprehensive understanding of the evolutionary dynamics and adaptive potential within a One Health context.

## Conclusions

In summary, our findings provide the first genomic characterization of *S. nepalensis* isolated from an anthropogenically impacted hypersaline lagoon in Brazil, revealing its presence and persistence in South America for at least four years prior to its first reported detection in the country. The clonal relationship between COLB and AM1 confirms the persistence and environmental stability of this lineage, while the acquisition of additional resistance determinants in AM1 highlights the dynamic genomic evolution occurring within an ecological niche.

The presence of Sal(E) across all 36 genomes supported by conserved chromosomal synteny, high amino acid identity, and structural homology to other ABC-F proteins, consistent with a vertically inherited species-associated determinant.

Collectively, the predominantly clonal population structure, open pangenome, and progressive acquisition of clinically significant resistance genes, including *mecA* in human clinical isolates from Viet Nam, underscore the potential emerging One Health relevance of *S. nepalensis* and highlight the critical need for accurate taxonomic identification and integrated genomic surveillance at the human-animal-environment interface.

## Supplementary Information

Below is the link to the electronic supplementary material.

Supplementary Material 1

Supplementary Material 2

Supplementary Material 3

Supplementary Material 4

## Data Availability

The whole-genome assemblies generated in this study have been deposited in GenBank under the following accession numbers: COLB (GenBank accession number: JBDFMR000000000.1) and AM1 (GenBank accession number: JBEGDQ000000000.1). The complete bioinformatics pipeline is available at https://github.com/zbecerra/Phylogeographic-analysis-of-S.-nepalensis-carrying-the-intrinsic-sal-E-resistance-gene.
