# Supplementary material for: Phylogeographic analysis of Staphylococcus nepalensis reveals global occurrence of antimicrobial-resistant lineages carrying the sal(E) resistance gene
Source: Mol Biol Rep. 2026 Sep 4;53(1):1525. doi: 10.1007/s11033-026-12601-4 (PMC13545133; doi:10.1007/s11033-026-12601-4)
Supplement: Supplementary file 1 [file 11033_2026_12601_MOESM3_ESM.pdf]

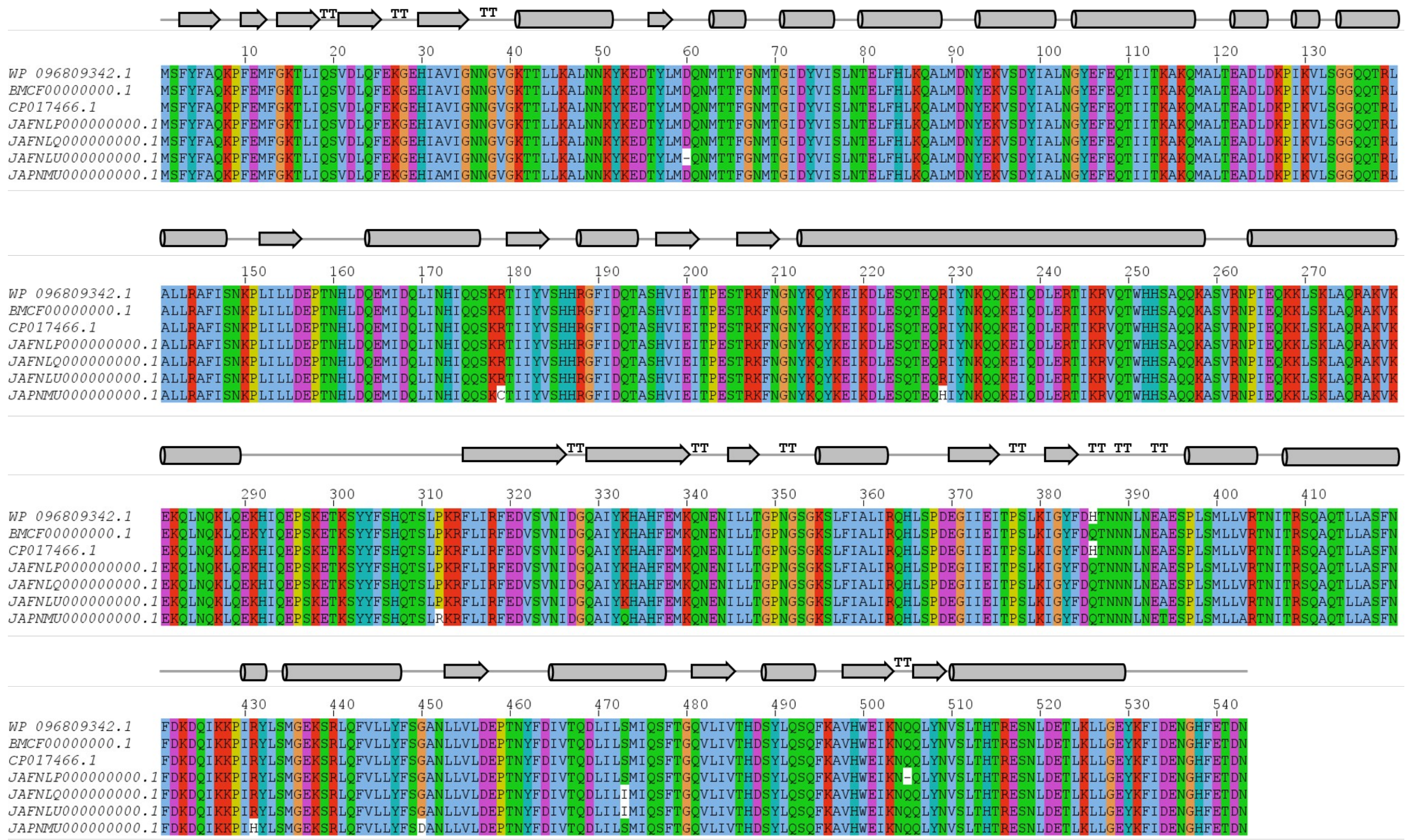

Fig. S1. Sequence alignment and structural annotation of representative Sal(E) ABC-F ribosomal protection proteins. On top of aligned sequences, α-helix (cylinders) and β-pleated sheets (arrows) of Sal(E) (GenBank: WP_096809342.1) were predicted in AlphaFold3. Representative sequences (GenBank: BMCF00000000.1, CP017466.1, JAFNLP000000000.1, JAFNLQ000000000.1, JAFNLU000000000.1, JAPNMU000000000.1) were selected via CD-HIT clustering based on amino acid identity with the reference sequence. Mutations relative to the reference are highlighted in white.
