# Supplementary material for: Phylogeographic analysis of Staphylococcus nepalensis reveals global occurrence of antimicrobial-resistant lineages carrying the sal(E) resistance gene
Source: Mol Biol Rep. 2026 Sep 4;53(1):1525. doi: 10.1007/s11033-026-12601-4 (PMC13545133; doi:10.1007/s11033-026-12601-4)
Supplement: Supplementary file 2 [file 11033_2026_12601_MOESM4_ESM.pdf]

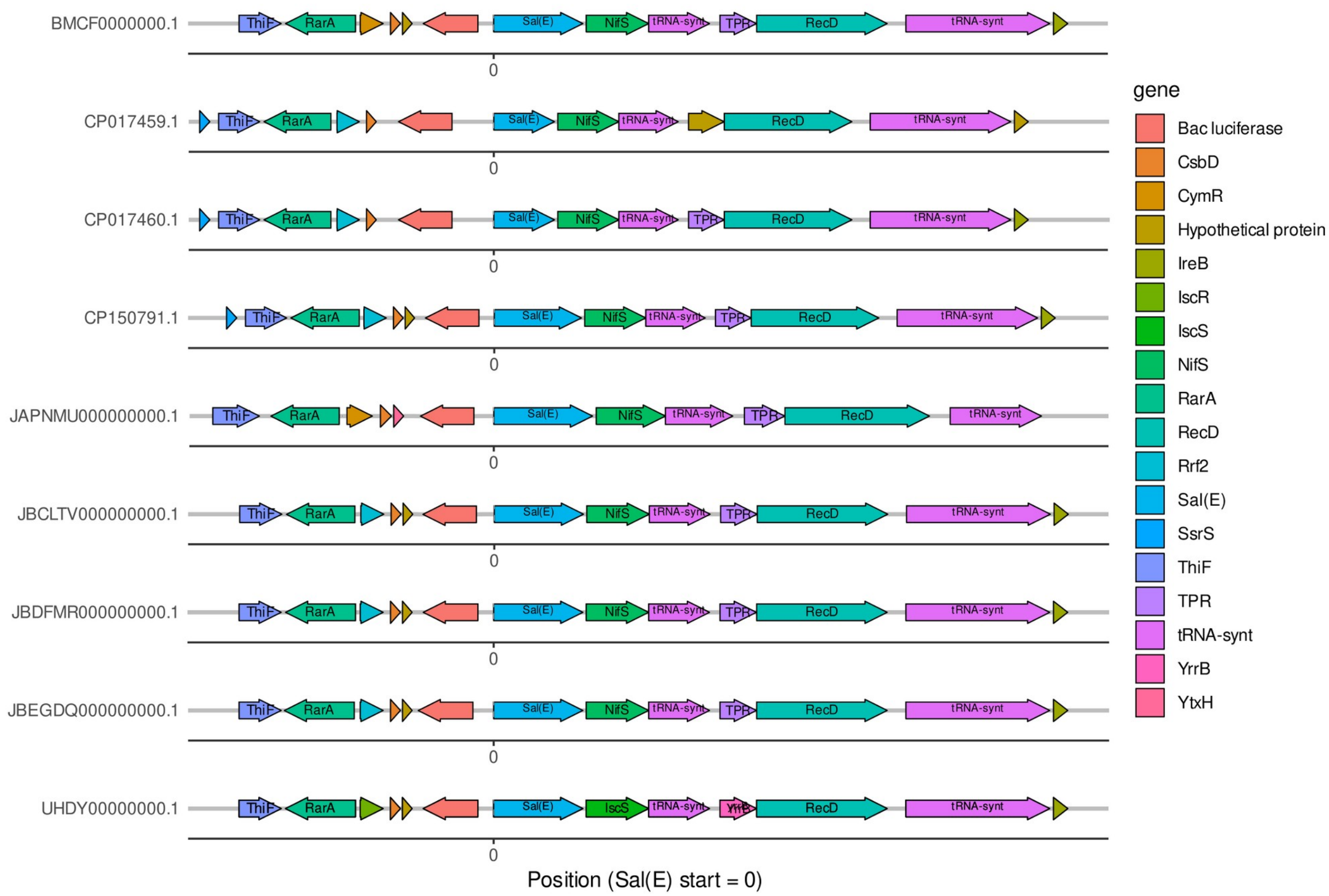

Fig. S2. Genetic context of the *sal*(E)-encoding ABC-F ribosomal protection protein (blue) in representative genomes analyzed in this study. Arrows represent coding sequences (CDSs), with arrow orientation indicating the direction of transcription, and the predicted functional categories denoted by color.
